# Arduino allows development of a low-cost, high-resolution metabolic and behavioral phenotyping system for birds

**DOI:** 10.1242/jeb.249921

**Published:** 2025-05-09

**Authors:** Elizabeth J. Rogers, Cory Elowe, Maria Stager, Alexander R. Gerson

**Affiliations:** ^1^ Organismic and Evolutionary Biology Graduate Program, University of Massachusetts, Amherst, MA 01003, USA; ^2^ Department of Biology, University of Massachusetts, Amherst, MA 01003, USA; ^3^ Department of Biology, Northern Michigan University, Marquette, MI 49855, USA

**Keywords:** Arduino, Metabolic phenotyping, Respirometry, Cold acclimation

## Abstract

Technological advancements now enable the use of flow-through respirometry for rapid, high-throughput metabolic phenotyping, but live-in systems currently do not exist for birds. We designed live-in respirometry chambers for small birds with the open-source electronics platform Arduino to continuously monitor bird body weight, food intake and water intake in sync with metabolic data collection. To demonstrate how this system can be implemented, we kept birds in the metabolic phenotypic chambers for 10 days while we progressively lowered the temperature from 25°C to 5°C. We used the data to calculate hourly energy expenditure and food/water intake during acute cold acclimation. We provide all plans and code for the live-in chambers, Arduino biomonitoring system and additional radio-frequency identification (RFID) module as a low-cost, DIY alternative to commercially available systems and to enable the use of standard respirometry equipment for metabolic phenotyping in birds.

## Teaser

**Summary:** DIY live-in chambers and Arduino electronics allow the use of standard respirometry equipment for high-resolution, multi-day metabolic phenotyping and bio-monitoring of birds.

## INTRODUCTION

Metabolic rate, the rate at which fuel is converted to energy or heat, is a central physiological trait that governs biological processes at all levels of organization (Brown et al., 2004). Intra- and interspecific variation in metabolic rate is of particular interest to eco-physiologists who seek to understand how animals respond to environmental stressors to better predict how animals may cope with rapid environmental change. Measuring whole-animal metabolism via indirect calorimetry by measuring rates of oxygen consumption and carbon dioxide production during aerobic respiration is a core technique for organismal physiologists, but accurate measurement of metabolism is complicated by the degree to which metabolic rate fluctuates with stress, circadian rhythms, exercise, thermoregulation, digestion, activity and many other factors (McKechnie and Swanson, 2010). To standardize measurements, physiologists tend to use a ‘snapshot’ approach where either minimal or maximal metabolic rates during relatively short time periods are taken from a longer period of metabolic measurement, presumably reflecting metabolism when the animal is in a constant state (e.g. Bartholomew and Dawson, 1953; Dawson and Dawson, 1982; Kleiber, 1961). Although minimal and maximal metabolic rates are useful measurements for making standardized comparisons within and among species, they assume conditions that animals rarely operate under in the wild and are difficult to extrapolate to real-world energy expenditure (Speakman et al., 1993).

One way to capture daily changes in metabolic rate is to measure oxygen consumption in wild, free-living animals by outfitting feeders or dwellings with respirometry equipment (Bartholomew and Lighton, 1986; Speers-Roesch et al., 2018; Tøien et al., 2015; Wolf et al., 2025). Field measurements, however, are limited to certain activities or seasonal periods (e.g. feeding or winter dormancy), may have to be averaged across multiple individuals sharing a cavity and are only suitable for taxa that will willingly use a feeder or enclosed dwelling. Multiday captive respirometry avoids many of these pitfalls and allows for greater control over extrinsic influences on metabolism, but the ability to collect high-resolution respirometry data is challenged by limitations on chamber volume, the rate of air flow through the system, and gas analyzer sensitivity and response rates (Lighton, 2017).

When conducting flow-through respirometry, the flow rate and chamber volume determine the amount of time it takes for the air in the chamber to turnover (i.e. the time constant), which determines the responsiveness of the system to changes in the metabolic rate of an animal. Slow time constants integrate metabolic signals over long time periods, precluding the ability to detect rapid changes in metabolism and historically, gas analyzers required relatively low flow rates (∼100–500 ml min^−1^) to detect changes in gas concentrations. To minimize the system's time constant, researchers then needed to keep respirometry chambers as small as possible, which severely limited the duration of time animals could safely be kept in the system.

Therefore, using respirometry for multi-day metabolic phenotyping first required several technological advancements which have been described in detail elsewhere (Lighton, 2017, 2019) and are summarized here. First, live-in respirometry chambers are required so animals can be provided with food and water, allowing metabolic measurements to be collected over multiple days. The loss in temporal resolution incurred from using relatively larger chambers needs then to be offset by higher flow rates and gas analyzers with high enough analytical sensitivity to detect very small fractional changes in gas concentrations. Commercial O_2_ analyzers are typically much less sensitive than CO_2_ analyzers and thus tend to be the limiting factor on a system's resolution. Additionally, as long as the excurrent air is well mixed, the response speed of most respirometry systems can be improved mathematically using an instantaneous correction (e.g. z-transformation, Bartholomew et al., 1981) which uses the derivative of the exponential O_2_ washout curve to predict instantaneous *V̇*_O_2__ (Lighton, 2019). Finally, resolution can be improved by measuring water vapor pressure so that gas concentrations can be mathematically corrected for the presence of H_2_O instead of using desiccant columns to chemically scrub water from incurrent air. Mathematical correction is preferred for high-throughput respirometry as chemical desiccant columns have a short lifespan, further increase the time constant of the system by increasing total volume and mix excurrent air in the column (Lighton and Halsey, 2011).

Multi-chamber respirometry systems, in which a multiplexer directs excurrent air to the gas analyzers and switches automatically between chambers, have the additional challenge of non-continuous sampling. The time saved by collecting data on multiple animals at once trades off with the potential loss of detail and overestimation of minimum metabolic rates that can occur when sampling is intermittent. Increasing sampling frequency by minimizing the length of each sampling interval increases the accuracy of interpolated measurements but depends on rapid response times of the gas analyzers. However, and to the advantage of the researcher in many instances, sampling more frequently than the time constant does not improve the resolution of metabolic measurements, but it can help average out noise introduced by high flow rates (Lighton and Halsey, 2011). The solution for enabling rapid multiplexing and increased chamber volume is therefore the same: decreasing the time constant by using high flow rates and highly sensitive gas analyzers.

These technological advancements have allowed the development of live-in, rapid-throughput respirometry systems that permit multiday collection of high-resolution metabolic data (herein called ‘metabolic phenotyping’) on multiple animals at once. Commercial live-in systems such as the OxyMax (Columbus Instruments International, Columbus, OH) and Promethion (Sable Systems International, Las Vegas, NV) also contain activity sensors with the ability to monitor eating, drinking and body mass, allowing behavior to be linked with changes in metabolic rate. Without electronic systems, the rate at which traits like food and water intake, body mass or body temperature can be monitored is constrained to the frequency at which researchers can make manual measurements without causing undue stress to the animal. While metabolic phenotyping is on the leading edge of metabolic research in the fields of human health and physiology (Rozman et al., 2014), these systems have yet to be widely adopted by eco-physiologists as they are not compatible with most non-model taxa, are often cost prohibitive and cannot be easily modified to monitor different traits or behaviors. To enable more widespread use of flow-through respirometry for multiday metabolic and behavioral phenotyping of small, endothermic vertebrates, we designed live-in respirometry chambers and a modular, DIY electronic bio-monitoring system that can be built in-house for a fraction of the cost of commercial systems. While designed for small birds, the system can be easily modified for different taxa or functionalities and can be used with multiple respirometry configurations.

Our custom-built metabolic phenotyping system uses commercially available gas analyzers and standard respirometry equipment alongside custom built live-in respirometry chambers outfitted with an Arduino module that we developed to collect continuous mass, food and water intake data. Arduino is an open-source platform popular among hobbyists used to build electronic systems controlled by simple, programmable circuit boards (i.e. microcontrollers). When we initially developed it in 2021, our Arduino module cost about US$115 per unit to produce; the most expensive components were the Arduino Uno (∼$15), the SD card data logger (∼$15), the load cells (∼$6 each) and load cell amplifiers (∼$10 each) and the real-time clock (∼$5). Arduino systems are fully customizable, and many different manufacturers produce Arduino-compatible electronic components, allowing flexibility in the cost, quality and functionality of the system. For the respirometry system, we made 13 liter live-in chambers from two sheets of ∼1 cm Plexiglass laser-cut to specification with 3D-printed components for holding the food and water. To maximize resolution of respirometry data, we used an O_2_ analyzer with a response time (10–90% of a step change) of <7 s and high sample flow rates (1.7–2 liters min^−1^) which when multiplexed, allowed us to sample each chamber for 60 s every 8 min. Full details and sources for components and assembly are available on Zenodo, an open-access online repository (doi:10.5281/zenodo.14827862). To demonstrate how this system can be implemented, we conducted metabolic phenotyping on house sparrows (*Passer domesticus*) for 10 days while progressively lowering the temperature of the chambers from 25°C to 5°C. The outcome provided proof-of-concept that we can use a combination of commercial respirometry equipment with DIY Arduino electronics to conduct metabolic and behavioral monitoring at a scale and resolution that has not been previously achieved for small passerines.

## MATERIALS AND METHODS

### Animal care

We captured house sparrows (*Passer domesticus*) for a larger experiment in October 2021 and May 2022 using mist nets and potter traps near the University of Massachusetts, Amherst campus (Amherst, MA, USA). To demonstrate the efficacy of the metabolic phenotyping system, we present a subset of the data collected from *n*=3 birds here. We moved birds to a University animal care facility within 1 h of capture and gave each individual unique color bands. Prior to starting the experiment, we kept birds in groups of 2–3 in cages (77.5×30.5×39 cm) with *ad libitum* water and a diet of ground Mazuri Small Bird Diet (Mazuri Exotic Animal Nutrition, St Louis, MO, USA) mixed with white millet seed. We kept birds on a 12 h:12 h light:dark cycle for the duration of the experiment.

Additional data collected from a single dark-eyed junco [slate-colored, *Junco hyemalis hyemalis* (Linnaeus 1758)] are presented here. This bird was captured in April 2024 in Amherst, MA, USA, and housed singly in a 30x40x40 cm cage with *ad libitum* seed (3:1 ratio of millet to black oil sunflower seed), vitamin fortified water and approximately 10 mealworms (*Tenebrio* sp.) daily. For this bird, core body temperature was measured using a temperature-sensitive Passive Integrative Transponder (PIT) tag (BioTherm13, BioMark, Boise, ID, USA). After at least 30 days in captivity and 20 days prior to metabolic phenotyping, a sterile PIT tag was injected subcutaneously above the scapulae using a large-gauge sterile needle (BioMark N125 PIT Tag Implanter Needle, 12G) and the injection site was sealed with adhesive glue (3M Vetbond Tissue Adhesive; Nicolaus et al. 2008).

The University of Massachusetts, Amherst Institutional Animal Care and Use Committee approved all procedures (protocols #2825 and #5274).

### Experimental procedure

**Fig. 1.**
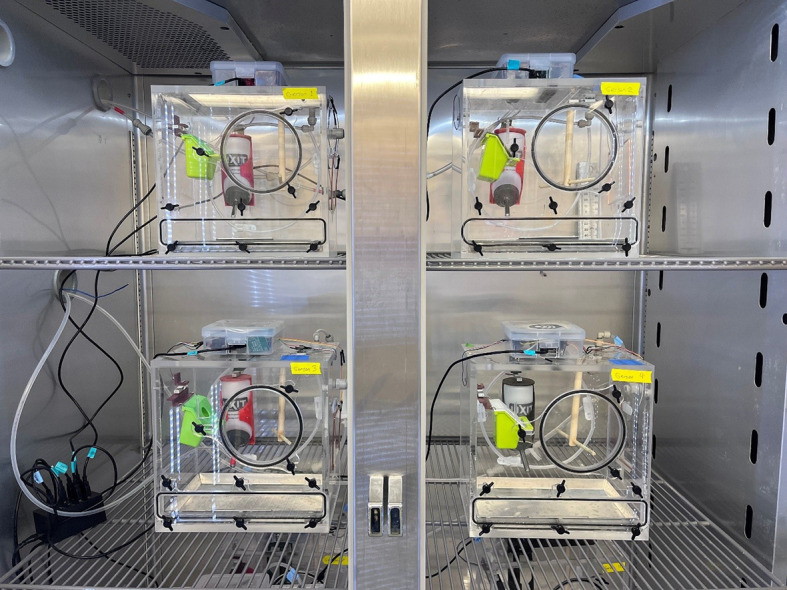
**Live-in bird metabolic phenotyping chambers are placed in a temperature-controlled environmental chamber.** Each 13 liters metabolic phenotyping chamber (27.5×27.5×17.5 cm) contains a perch, food dish and water dish connected to load cell sensors. The load cells are connected to Arduino microcontrollers in the boxes on top of the chambers which record the weight of the perch, food, and water every second. Gas is pulled from each chamber to an SSI respirometry system through Bev-a-Line tubing using individual pumps (outside the environmental chamber). The Arduino systems are powered using a multi-port 5 V USB power block (bottom left).

**Fig. 2.**
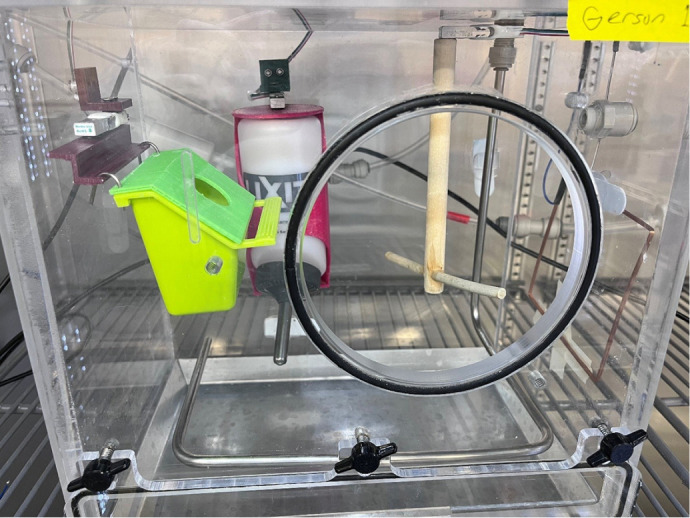
**Inside view of the metabolic phenotyping chamber.** The perch, food dish and water bottle are mounted to load cell sensors which are secured to the chamber on one end allowing the load cell to flex slightly when weight is added. The Arduino microcontroller (not pictured) is programed to convert the tension force detected by the load cell to a weight measurement. Air is continuously pulled from the chamber through a perforated steel tube (shown here) or Bev-a-Line tube (not shown) for respirometry. For this version of the chambers, an RFID antenna was mounted on the right side of the chamber to detect temperature-sensitive PIT tags within ∼13 cm.

After at least 2 weeks of acclimation to captivity, we moved birds (body mass=27.0±1.5 g; mean±s.d.) to individual live-in metabolic phenotyping chambers in a temperature controlled environmental chamber (Fig. 1; KB055, Darwin Chambers, St Louis, MO, USA). As sparrows discard the hulls of millet seed, we switched birds to a diet of only ground Mazuri Small Bird Diet to minimize error in food measurements. We also 3D printed food dish covers with a central 2.5 cm diameter hole to minimize food spillage (Fig. 2; see doi:10.5281/zenodo.14827862 for 3D files). We kept birds in the chambers for a total of 42 days; over a 10-day period after 3 weeks in the chambers, we ramped the temperature of the environmental chamber down gradually from 25°C to 5°C. During this time, we continuously monitored metabolic rate, body weight, and food and water consumption. We recorded temperature (°C) and relative humidity (RH, %) in the environmental chamber throughout using a Hobo logger (MX1104, Onset, Bourne, MA, USA).

### Metabolic phenotyping chambers

We custom built 27.5×27.5×17.5 cm (13 liter) chambers using 0.64 or 1.27 cm (¼″ or ½″) clear acrylic (see doi:10.5281/zenodo.14827862 for designs and laser files). The chambers include a 12.5 cm diameter access port at the center of the front panel and a 26×3.5 cm rectangular port at the bottom used to remove/insert an aluminium floor tray (Fig. 1). One chamber can be cut from two 24×36″ (∼60×90 cm) acrylic sheets using a laser-cutter and assembled with acrylic cement (Weld-on #4) and 90 deg angle clamps. Each port is outfitted with an acrylic door that is secured using plastic or stainless steel thumb screws. We installed a ¼″ bulkhead union adapter (John Guest, Yiewsley, UK) in the roof of the chamber to connect to the respirometry system with ¼″ Bev-A-Line tubing from the outside and ¼″ stainless steel tubing on the inside. To promote air mixing and even sampling, we drilled holes in the stainless steel tubing spaced 5 cm apart and bent the tubing so that it would surround the perimeter of the chamber approximately 5 cm from the bottom (Fig. 2). Bev-a-Line tubing can be used in lieu of steel tubing and secured to the back wall of the chamber (Fig. 1). We drilled two additional 2 cm ventilation holes on the top and side of the chamber and confirmed that the chambers were leaky enough such that the concentration of CO_2_ in the chamber stayed well below 2% if, in the event of a power outage or other unlikely event, the pumps stopped working.

To monitor the bird's body mass, we connected a 780 g micro load cell (Phidgets, Calgary, Canada) to a perch made from 0.6 cm wood doweling and mounted the load cell on the roof of the chamber using M3 cap screws and 1/8″ O-rings (Fig. 2). Using 3D printed brackets and mounts (see doi:10.5281/zenodo.14827862), we also connected food dishes (BPV1263, Prevue Pet Products, Chicago, IL) and water bottles [8 oz (∼250 ml) wide mouth, Lixit, Napa, CA] to 780 g micro load cells and mounted them on the walls of the chamber (Fig. 2). We connected each load cell to a load cell amplifier (HX711, Sparkfun, Boulder, CO), which was then connected to an Arduino Uno R3 board (Arduino, Turin, Italy). We also connected an OpenLog data logger (DEV-13712, Sparkfun, Boulder, CO) with a 32GB microSD card (SanDisk, Milpitas, CA) and battery-backed real time clock (RTC; PCF8523, Adafruit, New York, NY) to the Arduino. To circumvent the need for an external data logger and RTC, the chambers can be connected directly to a computer using an externally powered USB hub (HB-BUP7, Sabrent, Fort Lauderdale, FL) and set to log data in real-time using a data collection terminal software such as CoolTerm v. 2.2.0 (http://freeware.the-meiers.org). We installed female header pins on the breakout boards (i.e. load cell amplifiers, data logger and RTC) and connected everything using male–male jumper wires (see doi:10.5281/zenodo.14827862 for schematic). Arduino Unos require a 5 V supply and can be powered via a USB connection or a USB AC/DC charger.

Using Arduino IDE (v. 1.8.16), we calibrated the load cells using 25–200 g weights and programmed the Arduinos to record the weight on each load cell and a timestamp to a text file on the SD card every 1 s. We tared the load cells by resetting the systems every 2–3 days while the bird was off the perch. To reduce noise in the data, we calculated a rolling mean and standard deviation with a window size of 60 s for bird body mass, food mass and water mass. For all variables, we then selected the rolling mean with the lowest rolling standard deviation every 10 min. For all variables, we calculated the difference between each measurement and the next, propagated the error, and removed data points greater than 3 standard deviations from the group mean to ensure data were taken from stable periods of measurement. This filtering reduced the initial number of data points by three orders of magnitude, greatly reducing the processing power required for analysis. Other users may choose to use less stringent filters to retain more data points. Using the lag differences, we filtered out instances when the bird was perched on the food dish or water bottle and when the food or water was refilled (|Δfood| or |Δwater| >20). We found that some birds preferred to sleep on the food dish or water bottle; to rescue some body mass data, we extracted Δfood and Δwater values that had an absolute value between 20 g and 35 g and recoded them as body mass measurements. We calculated hourly and daily food and water consumption by summing Δfood and Δwater per hour; we calculated hourly and daily body mass by taking the average.

### Respirometry

Concentrations of O_2_, CO_2_, and H_2_O gas were monitored using an open flow-through respirometry system from Sable Systems International (SSI). We assessed up to 6 birds at once plus one empty respirometry chamber with a full water bottle and food dish. Ambient air in the environmental chamber was analyzed as a baseline. We calibrated the oxygen sensor (FC-10, SSI, Las Vegas, NV) using dry O_2_ tank gas of a known concentration (21.02±2%; Airgas, Randor, PA), and span calibrated the CO_2_ sensor (LI-840A, LI-COR Biosciences, Lincoln, NE) and zeroed H_2_O using dry CO_2_ tank gas (1.004±2%; Airgas, Randor, PA).

We pulled air from each chamber at rates between 1700 and 2000 ml min^−1^ using 20 p.s.i. (∼140 kPa) diaphragm pumps (GAST, Benton Harbor, MI) and adjustable AC/DC power adapters. We monitored flow rate using an SSI FlowBar-8, which automatically compensates for temperature and pressure. We placed in-line filters between the chamber and the respirometry system. At a chamber volume of 13 liters and a flow rate of 1700 ml min^−1^, 63% of the air in the chamber was replaced every 7.5 min (i.e. one time constant; Lighton, 2019). We multiplexed airstreams using an RM-8 Flow Multiplexer and subsampled air from each chamber sequentially for 60 s at 700 ml min^−1^ with an SS-4 Sub-Sampler (SSI, Las Vegas, NV). We made a baseline measurement for 60 s in between each cycle through the 7 chambers, resulting in one measurement per animal every 8 min (see doi:10.5281/zenodo.14827862 for plumbing diagram).

We used Expedata software (SSI, Las Vegas, NV, USA) to process the raw respirometry data (macro available on doi:10.5281/zenodo.14827862). The macro first calculated fractional concentrations of O_2_, CO_2_ and H_2_O with a z-transformation for O_2_ (Bartholomew et al., 1981; Lighton, 2019), lag correction (O_2_=7 s lag, CO_2_/H_2_O=5 s lag), and smoothing function. The second step corrected O_2_ concentration and flow rate for the presence of water vapor and baselined all gas measurements relative to ambient across the most stable 50% of the final 30 s of each sampling window (Colella et al., 2021). Finally, the macro calculated *V̇*_O_2__ (ml min^−1^), *V̇*_CO_2__ (ml min^−1^), *V̇*_H_2_O_ (mg min^−1^) using standard equations for pull respirometry (Lighton, 2019). We calculated hourly and daily gas exchange values by calculating the area under the curve and used total O_2_ and CO_2_ to calculate hourly and daily energy expenditure (EE, kJ) using the Weir formula (Weir, 1949). We calculated minimum daily metabolic rates by taking the average of the two lowest *V̇*_O_2__ measurements each day (24 h). We report uncorrected *V̇*_H_2_O_, which we assume primarily represents respiratory and cutaneous water loss with small, unquantified, inputs from the evaporation of fecal/urinary water and spilled drinking water.

### Data analysis

We used linear models to investigate changes in total hourly EE, *V̇*_H_2_O_, and food and water consumption; in all cases we selected the most appropriate combination of predictor variables by choosing the model with the lowest AIC. Hourly average *V̇*_H_2_O_ and hourly water consumption were both right skewed, so we log transformed the variables prior to analysis. The starting model for hourly energy expenditure (EE) included mean temperature (°C), hour and bird ID. The starting model for *V̇*_H_2_O_ included mean temperature (°C), mean relative humidity (%), hour and bird ID. To analyze the effects of EE on hourly food and water consumption, we filtered out data points from nighttime inactive hours. The starting model for hourly food consumption included hourly EE, hour and bird ID. For hourly water consumption, we tested the effects of hourly EE, hourly average *V̇*_H_2_O_, hour and bird ID.

## RESULTS AND DISCUSSION

**Fig. 3.**
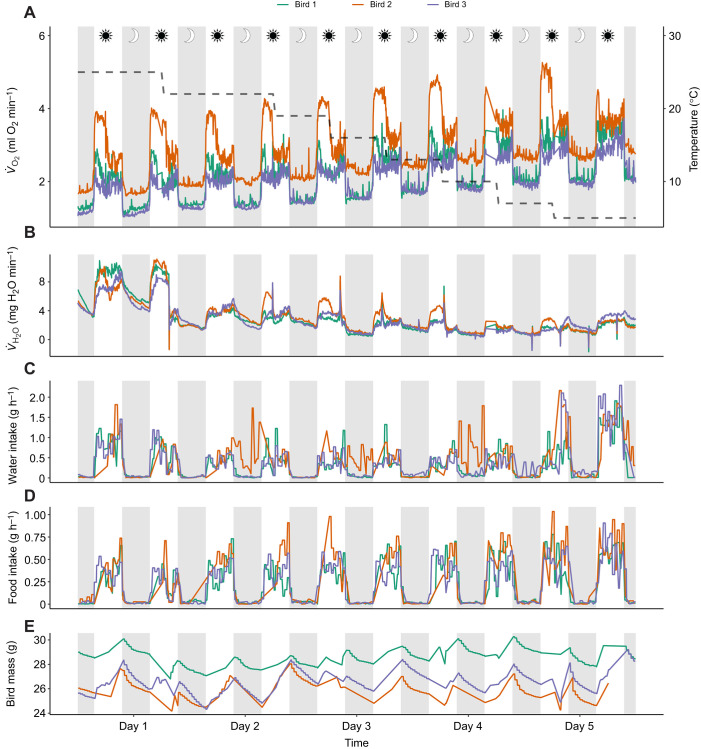
**Time series showing temperature, RMR, *V̇*_H_2_O_, water intake, food consumption and bird body mass for the duration of the experiment.** (A) Temperature (dashed line) and RMR, (B) *V̇*_H_2_O_, (C) water intake, (D) food consumption and (E) bird body mass for three individual house sparrows. Respirometry data (A,B: RMR and *V̇*_H_2_O_) include one data point per time constant (7.5 min) and Arduino data (C–E: water, food and body mass) include one data point per hour. Each line represents one bird. Gray rectangles indicate night (lights off) and white rectangles indicate day (lights on).

The ability to accurately measure components of animal metabolism is crucial for determining an animal's physiological requirements and limits to survival. We demonstrate here the ability to collect high-resolution metabolic and behavioral data for rapid metabolic phenotyping using a pull respirometry system and low-cost DIY Arduino sensors. During 10 days of data collection, we recorded 1643 60 s intervals of gas exchange data and 792,549 data points from the Arduinos per load cell, per bird. After processing the Arduino data and removing outliers, we retained an average of 853 water measurements, 725 food measurements and 742 body mass measurements per bird (Fig. 3C–E). The data collected from the Arduinos fell within the expected ranges of body mass and daily food and water consumption for sparrows in captivity at 5–25°C (Bartholomew and Cade, 1963; Seibert, 1949). Bird mass ranged from 23.0 g to 30.3 g and averaged 27.0±0.3 g (mean±s.d.). Daily food intake ranged from 3.1 g to 8.9 g and averaged 5.7±3.6 g. Daily water intake ranged from 5.4 g to 20.8 g and averaged 9.4±0.04 g. We calculated daily drift (daily max.–daily min.) for each sensor in the empty chamber; the perch mass measurement drifted 0.5±0.4 g daily, while the full food and water sensors drifted 0.04±0.02 g and 0.05±0.03 g, respectively.

**Fig. 4.**
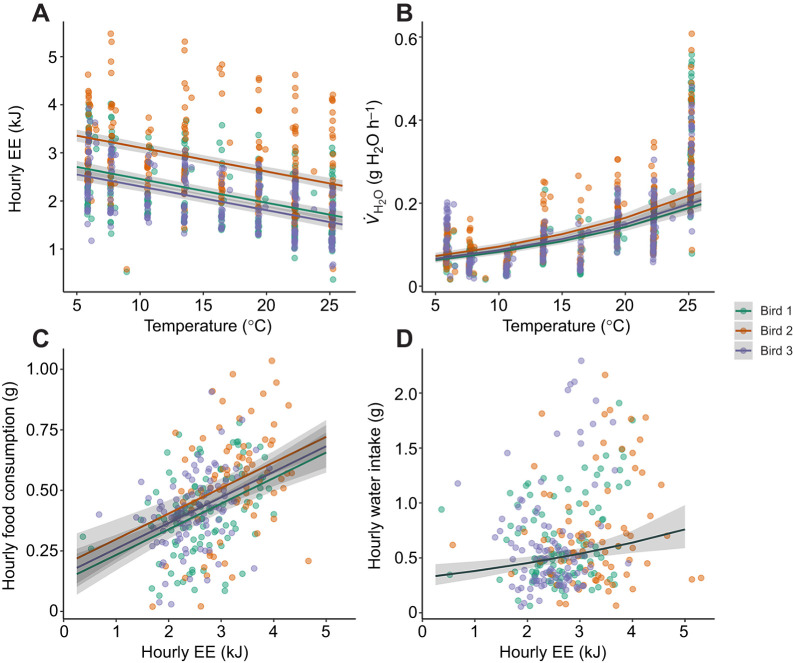
**Changes in hourly EE and *V̇***_**H_2_O**_ **with temperature (5–25°C) and hourly food consumption and water intake with EE.** (A) Hourly EE increases and (B) hourly *V̇*_H_2_O_ decreases logarithmically as the temperature in the environmental chamber declines. Hourly food consumption (C) and hourly water intake (D) increase with EE. Each bird is represented by a different color. Lines represent means and 95% confidence intervals from the model predictions and points represent the raw data.

By simultaneously collecting metabolic and behavioral data, we were able to tightly synchronize changes in EE, *V̇*_H_2_O_ and food/water intake with temperature as birds were exposed to progressively colder temperatures Fig. 3A–D. Average minimum metabolic rate at 25°C ranged from 1.0 to1.6 ml O_2_ min^−1^, which is consistent with previous measurements of basal metabolic rate (BMR) for house sparrows (Arens and Cooper, 2005b; Chappell et al., 1999). We found the best fit model for hourly EE included bird ID, hour and temperature as covariates (*F*_4,696_=86.5, *P*<0.0001) and that hourly EE increased 0.05 kJ per degree decrease in temperature (Fig. 4A; *F*_1,696_=162.0, *P*<0.0001). There was no breakpoint in EE suggestive of a thermoregulatory limit (Kleiber, 1961); reported lower critical temperatures (i.e. the temperature below which metabolism must increase to maintain constant body temperature) for house sparrows range from 20 to 25°C (Arens and Cooper, 2005a; Kendeigh, 1969; Nzama et al., 2010); thus it is likely birds fell below thermoneutrality following the first step down in temperature from 25°C to 22°C. Hourly *V̇*_H_2_O_, which was best modeled by bird ID, temperature, hour and relative humidity (RH; *F*_5,695_=153.2, *P*<0.0001), increased 5% per degree increase in temperature (Fig. 4B; *F*_1,695_=262.1, *P*<0.0001) and decreased 4% for each percentage increase in RH (*F*_1,695_=56.1, *P*<0.0001). The best fit model for hourly food included hourly EE and bird ID as covariates (*F*_3,270_=23.1, *P*<0.0001) while hourly water consumption was best modeled by hourly EE and hour (*F*_2,283_=7.5, *P*<0.001). Hourly food consumption increased 0.1 g with every kJ increase in hourly EE (Fig. 4C; *F*_1,270_=41.3, *P*<0.0001) and hourly water consumption increased 17% with every 1 kJ increase in hourly EE (Fig. 4D; *F*_1,305_=9.0, *P*=0.003).

We present these data as proof-of-concept and to demonstrate how Arduinos can be implemented alongside standard respirometry equipment to build a low-cost, novel metabolic phenotyping system for birds. While the chambers in this study were designed for birds, the load cells could easily be replaced with force sensors to accommodate different types of food and water dispensers. Additional flexibility in the Arduino software platform offers the possibility of using the same system for different applications; for example, the perches could be reprogrammed to monitor perching frequency as a proxy for bird activity. Arduino systems are endlessly customizable and many other uses of Arduino-based electronics have been documented for biological research including RFID-enabled nest boxes, a seawater pH control system, a cell stretcher and an LED stimulator system for vision research (Bridge et al., 2019; McLean et al., 2021; Ragazzini et al., 2021; Teikari et al., 2012).

**Fig. 5.**
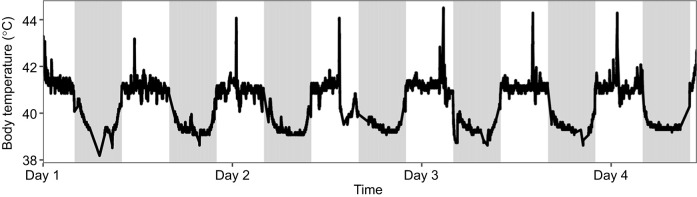
**Body temperature of a single dark-eyed junco (*Junco hyemalis hyemalis*) in a metabolic phenotyping chamber.** An RFID module with a 13 cm antenna range was added to the Arduino to read every 2 s for a temperature-sensitive PIT tag that was injected subcutaneously. Individual points depict body temperature readings when the bird was within range of the antenna.

To that end, in a second iteration of the system, we added an RFID module and antenna. We implanted a temperature-sensitive passive integrated transponder (PIT) tag in a single dark-eyed junco (*Junco hyemalis hyemalis*) and demonstrated continuous collection of body temperature data within the chamber (Fig. 5). We used an RFID module (see doi:10.5281/zenodo.14827862 for details) that integrates with the Arduino system and attached the included antenna to the outside of the chamber, enabling body temperature assays whenever the subcutaneously tagged bird was located on the perch or ∼13 cm from the side of the chamber with the antenna (Fig. 2). While this allowed for continuous monitoring of body temperature when the animals were within range of the antenna, a custom antenna may be able to increase coverage of the chamber for more frequent reads (Fig. 5).

Technological advancements are enabling novel methods for high-resolution data logging and new entryways into low-cost, do-it-yourself science. Arduino provides a modular and completely customizable platform for building automated electronic systems and we hope that the system presented here provides an example of how continuous biomonitoring can be implemented alongside rapid flow-through respirometry. The cost of each metabolic phenotyping chamber presented here was ∼US$230 per unit at the time of development, including the electronics and live-in chamber (but independent of the respirometry system), which is far below the cost of commercially available systems for rodents. The system requires more maintenance than commercially available systems; a few load cells and RTCs had to be replaced or recalibrated over the course of the experiment, and there was occasional malfunction of the SD card data loggers. In future iterations of this system, users may opt to use higher-quality electronics and may choose to modify the 3D printed components to prevent birds from sleeping on the food dishes and water bottles. Many of the potential issues with the RTCs and SD cards can also be avoided by logging data in real time. This can be accomplished with an externally powered USB hub that can both power the Arduinos and interface with a computer. Data streams from all the chambers can be read using a serial monitoring program (such as CoolTerm or RealTerm) and writing the data directly to a text file while adding time stamps directly from the computer.

In summary, we show how Arduino can be used to build a modular behavioral and metabolic phenotyping system for small birds, and we hope that other users will continue to improve upon this design to meet their own needs. Moreover, we hope that the materials provided here will allow others to replicate this system with minimal expertise required. For instance, many of the components should be able to be fabricated on college campuses or with the use of a Maker lab. While we don't intend for this system to replace commercially available metabolic phenotyping systems, it provides a low-cost, customizable alternative for non-model organisms.
